# Extending AAV Packaging Cargo through Dual Co-Transduction: Efficient Protein Trans-Splicing at Low Vector Doses

**DOI:** 10.3390/ijms241310524

**Published:** 2023-06-23

**Authors:** Mariana V. Ferreira, Sofia Fernandes, Ana Isabel Almeida, Salomé Neto, João P. Mendes, Ricardo J. S. Silva, Cristina Peixoto, Ana Sofia Coroadinha

**Affiliations:** 1 iBET-Instituto de Biologia Experimental e Tecnológica, Apartado 12, 2781-901 Oeiras, Portugal; mariana.ferreira@ibet.pt (M.V.F.); as.fernandes@ibet.pt (S.F.); anaa@ibet.pt (A.I.A.); salome.neto@ibet.pt (S.N.); joaopmendes10@gmail.com (J.P.M.); rsilva@ibet.pt (R.J.S.S.); peixoto@ibet.pt (C.P.); 2 Instituto de Tecnologia Química e Biológica António Xavier, Universidade Nova de Lisboa, Av. da República, 2780-157 Oeiras, Portugal

**Keywords:** dual AAV vector, split-inteins, full capsid enrichment, AAV co-transduction, dual AAV-intein mediated systems

## Abstract

Adeno-associated viral (AAV) vectors represent one of the leading platforms for gene delivery. Nevertheless, their small packaging capacity restricts their use for diseases requiring large-gene delivery. To overcome this, dual-AAV vector systems that rely on protein trans-splicing were developed, with the split-intein Npu DnaE among the most-used. However, the reconstitution efficiency of Npu DnaE is still insufficient, requiring higher vector doses. In this work, two split-inteins, Cfa and Gp41-1, with reportedly superior trans-splicing were evaluated in comparison with Npu DnaE by transient transfections and dual-AAV in vitro co-transductions. Both Cfa and Gp41-1 split-inteins enabled reconstitution rates that were over two-fold higher than Npu DnaE and 100% of protein reconstitution. The impact of different vector preparation qualities in split-intein performances was also evaluated in co-transduction assays. Higher-quality preparations increased split-inteins’ performances by three-fold when compared to low-quality preparations (60–75% vs. 20–30% full particles, respectively). Low-quality vector preparations were observed to limit split-gene reconstitutions by inhibiting co-transduction. We show that combining superior split-inteins with higher-quality vector preparations allowed vector doses to be decreased while maintaining high trans-splicing rates. These results show the potential of more-efficient protein-trans-splicing strategies in dual-AAV vector co-transduction, allowing the extension of its use to the delivery of larger therapeutic genes.

## 1. Introduction

Adeno-associated viral (AAV) vectors are currently the most well-established vectors in clinical trials for in vivo gene therapies. To date, Hemgenix^®^ [1] was the last AAV vector-based product approved and several others are currently under phase III clinical development [2,3]. Despite the immense potential of these vectors, their limited cargo capacity (∼5 kb) has restricted their use in delivering larger DNA sequences. Several common inherited diseases result from mutations in genes with a coding sequence (CDS) larger than 5 kb. If we consider all cis-regulatory elements that are required for cassette expression, the maximum CDS size further decreases to 3.5–4 kb [2].

Dual-AAV vectors, each carrying split fragments of the therapeutic gene, have been explored to overcome this limitation. The majority of dual-AAV vector strategies are based on DNA trans-splicing mechanisms such as ITR concatemerization or homologous recombination [4,5]. However, these approaches have not proven to be successful in all cases, as the CDS reconstitution is dependent on complex DNA repair pathways and can vary from different tissue/cell types, leading to diverse gene reconstitution rates [2,6]. Dual-AAV vector-intein-mediated delivery is an alternative approach, using protein trans-splicing to reconstitute large proteins [2,7].

Inteins are genetic elements that are inserted in-frame into protein-coding genes. Intein-containing genes can be split into two fragments and, subsequently, link their flanks by a protein trans-splicing reaction, generating a full-length mature protein. Each of the split genes codes for a separate polypeptide: one consisting of the N-terminal part of the protein of interest followed by the N-terminal part of the intein and the other consisting of the C-terminal part of the intein followed by the C-terminal part of the protein of interest. The two split-inteins are able to associate specifically with each other, leading to a protein-splicing reaction, which ligates the two protein parts in trans [8,9,10]. Protein-splicing is a robust and quick reaction, without the need of cofactors or external energy sources. This results in an attractive system for dual-AAV vector delivery, as protein-splicing has been shown to be more efficient than the previous strategies [11]. However, the design of dual-AAV vector-intein constructs is particularly challenging, as the splitting site of the therapeutic protein needs to be outside of structural domains to avoid incorrect protein folding and specific intein amino acid residues need to be present for efficient splicing [11]. In many cases, a few extein amino acid residues can remain after protein reconstitution, and these residues may affect protein function. Thus, a tailored screening of the protein-splitting sites is needed. Additionally, the size and sequence of the extein amino acids can be optimized. The best approach is highly dependent on the protein of interest. The split-intein sequence from *Nostoc Punctiforme* (Npu DnaE) is an extensively studied split-intein and is among the most-used in dual-AAV vector delivery [12,13,14]. Although it exhibits good reconstitution efficiencies, other split-inteins, such as engineered-consensus (Cfa) and cyanophage-like (Gp41-1), either synthetically engineered or naturally occurring, can achieve significantly superior reconstitution rates [15,16,17].

Currently, dual-AAV vector approaches have shown lower expression levels of the therapeutic gene in comparison with single-AAV vectors [4,18,19,20]. This limitation is mainly related to inefficient gene reconstitution. To circumvent this, dual-AAV vector-intein mediated strategies use high-vector doses of up to 10^11^ V.G./eye of each vector in vivo pre-clinical studies [4,19]. Nevertheless, this can raise important safety concerns, increase the probability of vector co-transduction competition, and increase overall higher-vector manufacture cost.

AAV vector production has been widely described as an imperfect process, creating heterogenic vector products with a different range of empty-to-full capsids. Low-quality vector productions with increased empty or defective capsids can limit transduction and lead to even higher adverse immunogenic effects [21,22,23,24]. The impact of vector quality on dual-AAV vector systems can be even more preeminent, as it can contribute to limiting gene reconstitutions by promoting vector competition and inhibiting co-transduction. Some downstream purification processes have been designed to overcome this, allowing the separation of empty and full AAV particles. Chromatography-based purification methods, such as anion exchange chromatography (AEX), offer a scalable and consistent option for full particle enrichment [25].

As dual-AAV vector-intein-mediated systems become more of standard practice in gene therapy, further studies and optimizations are crucial in extending the systems’ potential. This work aims to overcome inefficient protein reconstitution rates and decrease the vector doses of current systems by applying split-inteins (Cfa and Gp41-1) with improved trans-splicing rates, combined with higher-quality AAV vector preparations [26,27].

## 2. Results

**Figure 1:**
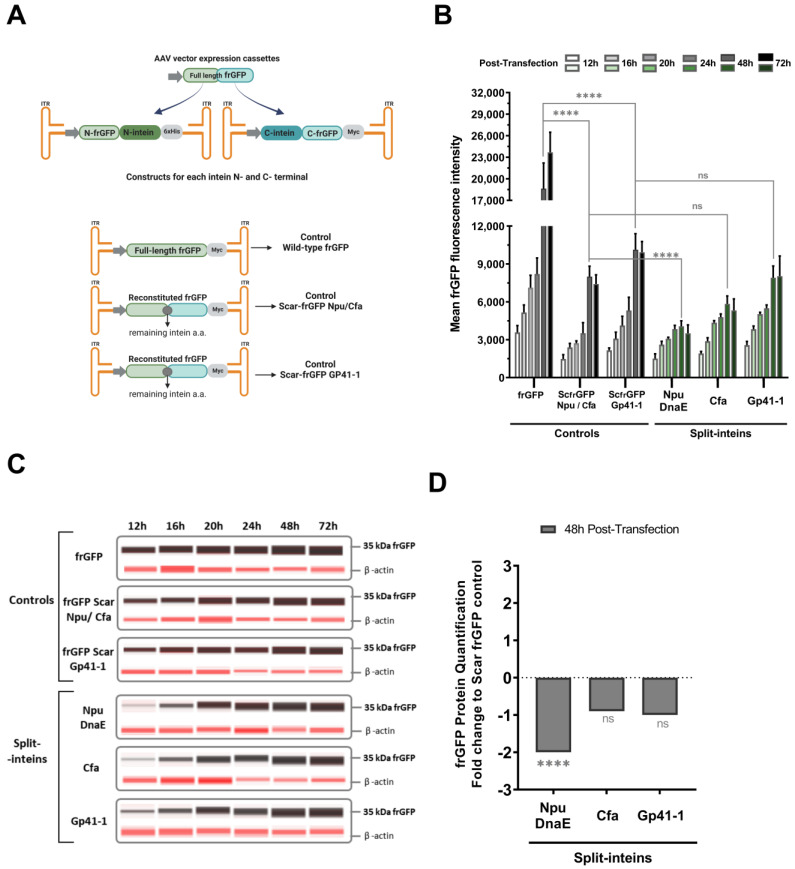
Split-inteins evaluation by transient transfection. (**A**) Schematic representation of the established AAV vector expression cassettes; all cassettes are between 4.1 to 4.3 k.bp. Split-intein constructs use a split reporter frGFP gene fused to the respective terminal of each split-intein. Three control AAV vector expression cassettes present a full-length frGFP gene, with or without the remaining extein amino acid residues (scars) generated upon protein trans-splicing. N-terminal constructs have a 6xHis-tag, and C-terminal and control constructs have a Myc-tag for Western blot detection. (**B**) frGFP protein reconstitution evaluation was performed by flow cytometry at different time points post-transfection. Fluorescence intensity values are shown as the mean of frGFP intensity. (**C**,**D**) Capillary Western blot analysis of frGFP protein reconstitution levels. Cell extracts were analyzed by immunoblotting with anti-Myc-tag antibody and anti-β-actin. Full-length or reconstituted frGFP is shown as a 35 kDa protein detected by chemiluminescence and β-actin protein with 47 kDa by fluorescence. (**C**) WB compass software 6.2.0 (ProteinSimple, CA, USA) was used for the simulation of a standard polyacrylamide gel run. (**D**) Quantification of split-inteins frGFP protein reconstitution. Values are shown as fold-change relative to the mean of frGFP protein quantified in the controls at 48 h post-transfection. frGFP: folding reporter GFP. ScfrGFP: scar frGFP. All data represent the mean ± SD; *n* = 3; ns—no significance; **** *p* < 0.0001 by Tukey’s post hoc multiple comparison test. The illustrations were created with BioRender.com accessed on the 4 April 2023.

Studies were conducted to evaluate the performances of split-inteins Cfa and Gp41-1 compared to that of the Npu DnaE. In all three of them, AAV vector expression cassettes were developed with a split folding reporter GFP (frGFP) [28,29] gene fused to the respective terminal of each split-intein (Figure 1A).

As previously mentioned, after protein reconstitution, some leftover amino acid residues remain and may affect its function. The split-intein amino acids (hereinafter designated as scars) left after split-intein protein trans-splicing are of known sequence, as they correspond to the N’ and C’ exteins used and required for ligation [27]. To correctly evaluate protein reconstitution, full-length frGFP controls were developed, namely a wild-type frGFP construct and frGFP constructs comprising the remaining scar amino acid residues left after split-intein reaction. Since Npu DnaE and Cfa split-inteins share the same extein residues, a scar-frGFP Npu/Cfa construct was established for each of them as a full-length control. Additionally, a second scar-frGFP Gp41-1 construct was developed as a direct control for the Gp41-1 split-intein (Figure 1A) [26,27]. The scar amino acid residues were confirmed to be present by peptide mapping of the final reconstituted proteins in cell extracts analyzed by mass spectrometry (Figure S1).

### 2.1. Evaluation of the Split-Inteins Trans-Splicing Performance by Transient Transfection

Initial evaluations of the split-intein protein reconstitution efficiencies were performed by transient transfection of the AAV vector expression cassettes. Protein reconstitution was evaluated at different time points post-transfection by flow cytometry and capillary Western blot.

The frGFP reconstitution was observed in more than 90% of the cells in all split-inteins tested, including in the earlier time point of 12 h post-transfection, and maintained over the time of the assay (Figure S2). Different fluorescence intensities were observed throughout the experimental conditions, allowing the evaluation of the split-inteins’ reconstitution efficiencies (Figure 1B). The results showed a two-fold decrease in fluorescent intensities in the frGFP scars controls when compared to wild-type frGFP control. Due to this finding, each split-intein was evaluated against the correspondent scar control for a correct protein reconstitution performance assessment. The use of split-inteins Cfa and Gp41-1 to generate full frGFP resulted in similar mean fluorescence intensity values when compared to the respective controls and two-fold higher values than when using Npu DnaE (Figure 1B). Capillary Western blot analyses of the cell extracts supported the flow cytometry results (Figure 1C,D), as similar levels of reconstituted frGFP total protein weredetected in Cfa and Gp41-1 split-inteins trans-splicing reaction samples. Moreover, up to two-fold lower reconstituted frGFP protein was quantified in the Npu DnaE samples when compared to the correspondent control. Regarding the control conditions, similar amounts of full-length frGFP protein were detected in all three (Figure 1C and Figure S3).

### 2.2. Evaluation of the Split-Inteins Trans-Splicing Performance by Dual-AAV2 Vector In Vitro Co-Transduction

#### 2.2.1. Assessment of Dual-AAV2 Vector Co-Transduction Doses

For a proper assessment of the split-inteins’ trans-splicing performances is crucial to determine the best co-transduction conditions. For that, a study was conducted to assess the efficiency of single vs. dual transductions at different vector multiplicities of infection (MOI).

**Figure 2:**
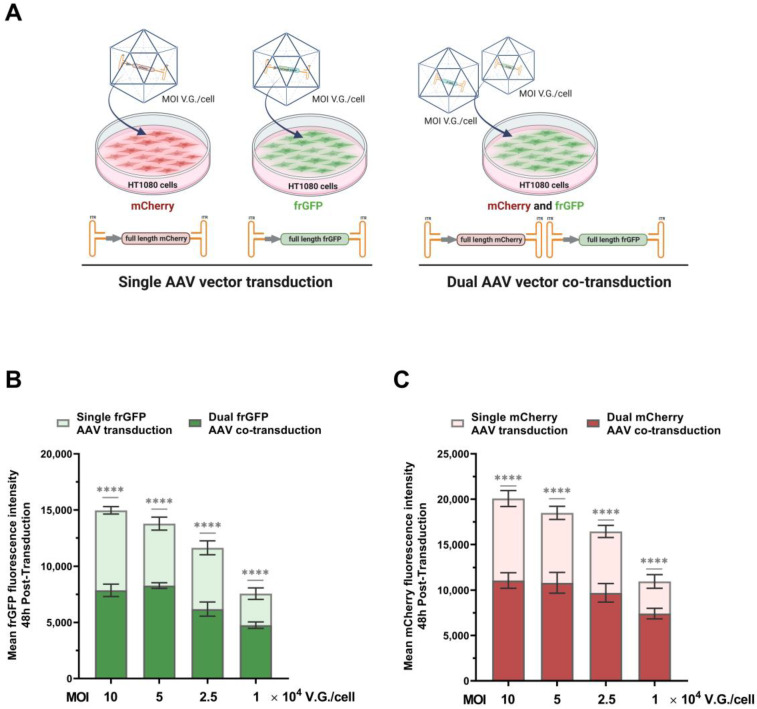
Evaluation of AAV2 vector single and dual co-transductions’ efficiencies at different vector doses. Four different vector doses (1 × 10^4^ to 1 × 10^5^ V.G./cell for each vector) were used to transduce HT1080 cells. Protein expression was assessed by flow cytometry at 48 h post-transduction. (**A**) Illustration of the transduction assay; on the left is a representation of the single transductions using either AAV vector expression constructs encoding for full-length mCherry or frGFP. On the right is the dual-AAV vector co-transductions, with each of the vectors encoding for mCherry and frGFP. (**B**,**C**) Fluorescence intensity values are shown as the mean of frGFP and mCherry intensities, respectively. Lighter bars represent single transductions with an average of 40% higher fluorescence intensities than those of dual-transductions shown by the darker bars. frGFP: folding reporter GFP. MOI: multiplicity of infection. All data represent the mean ± SD; *n* = 3; **** *p* < 0.0001 by Tukey’s post hoc multiple comparison test performed between single and dual co-transduction at the respective MOI. The illustrations were created with BioRender.com accessed on the 4 April 2023.

Therefore, single and dual co-transductions were performed using AAV2 vectors coding for wild-type frGFP and mCherry (Figure 2A). Several vector doses were tested and evaluated by flow cytometry, in line with what is described in the literature [2] (MOI: 1 × 10^4^ to 1 × 10^5^ V.G./cell for each vector). The results showed 100% transduction efficiencies (Figure S4) with increasing fluorescence intensities at higher vector doses (Figure 2B). An overall decrease of 40% in fluorescence intensity (up to two-fold reduction) in dual co-transduction was observed, compared to single co-transduction (Figure 2B). Moreover, above the MOI of 5 × 10^4^ V.G./cell fluorescence intensities did not increase in dual co-transduction, suggesting a signal saturation. Nevertheless, 5 × 10^4^ V.G./cell MOI was chosen for further studies, as it rendered the maximum transduction and, therefore, the most appropriate conditions for trans-splicing.

#### 2.2.2. Evaluation of Split-Inteins’ Trans-Splicing Efficiencies and Impact of AAV2 Vector Quality

The split-inteins’ trans-splicing efficiency was also assessed in the context of dual-AAV vector co-transduction. We questioned what would be the influence of empty particles to the co-transduction efficiencies and to the split-inteins’ trans-splicing performances. To answer to that question, an upstream-to-downstream process was performed with and without a full genome particle enrichment step (using anion exchange chromatography, as shown in Figure S5). Two different sets of AAV2 vector quality preparations for each split-intein under evaluation were obtained. Herein, we designated as lower quality the preparation obtained without full genome particles’ enrichment, which presented, on average, 20–30% of full AAV particles. We designated higher-quality preparations as the ones subjected to an additional step of full genome particles’ enrichment with an average of 60–75% of full AAV particles. Overall, the established AAV vector full particle enrichment step increased the quality of the viral preparations by three-fold.

**Figure 3:**
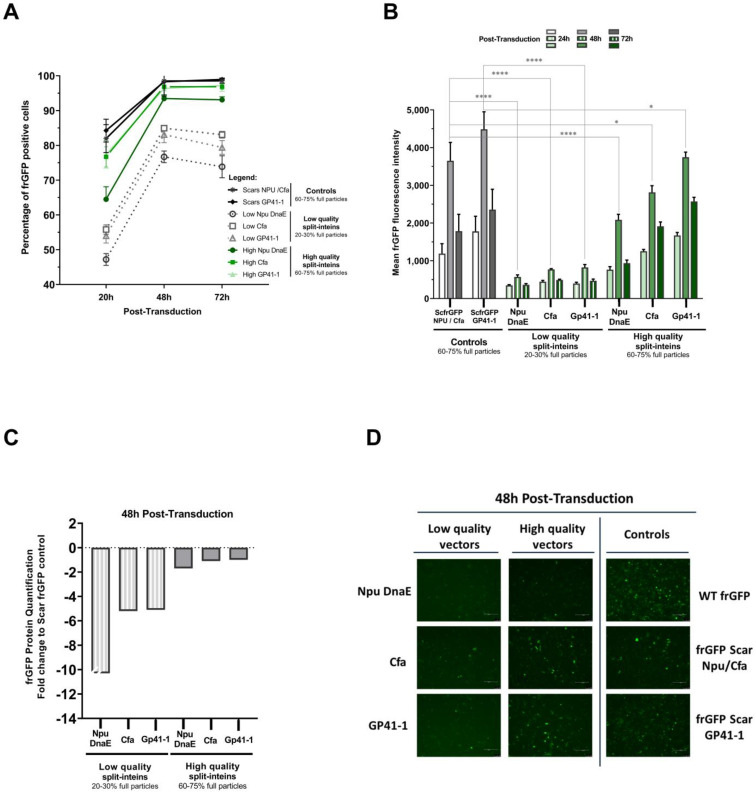
Impact of vector quality on split-inteins’ performances. Lower-quality preparations presented an average of 20–30% of full particles and higher-quality preparations presented an average of 60–75% of full particles. Lower- and higher-quality AAV2 vector split-intein preparations were used to co-transduce HT1080 cells with a vector dose of 5 × 10^4^ V.G./cell for each vector. The frGFP protein reconstitution evaluation was performed by flow cytometry at three different time points post-transduction. (**A**) The percentage of cells with reconstituted frGFP. The difference of 10–15% less transduced positive cells with lower-quality AAV2 vector preparations represents a significance of ****. (**B**) The reconstituted frGFP fluorescence intensity values, shown as the mean of frGFP intensity. Capillary Western blot analysis of frGFP protein reconstitution levels was performed for all time points (shown in Figure S6). Cell extracts were analyzed by immunoblotting with an anti-Myc-tag antibody. Full-length or reconstituted frGFP is shown as a 35 kDa protein. (**C**) Quantification of split-inteins frGFP protein reconstitution. Values are shown as fold-change relative to the mean of frGFP protein quantified in the controls at 48 h post-transduction. (**D**) Fluorescence images of frGFP protein expression upon transduction at 48 h (scale bar = 200 μm). frGFP: folding reporter GFP. ScfrGFP: scar frGFP. Flow cytometry data represent the mean ± SD; *n* = 3; * *p* ± 0.046, **** *p* < 0.0001 by Tukey’s post hoc multiple comparison test.

To assess the impact of AAV vector quality (i.e., the percentage of empty and full particles), both preparations were used for a co-transduction assay, first at an MOI of 5 × 10^4^ V.G./cell. Reconstitution of the frGFP protein was evaluated at different time points post-transduction by flow cytometry and capillary Western blot. Higher-quality split-intein preparations showed over 90% of transduced cells with frGFP reconstitution. In contrast, low-quality vector preparations presented only up to 75–85% of cells with frGFP reconstitution, a difference of 10–15% of transduced positive cells (Figure 3A). Additionally, an overall three-fold increase in fluorescence intensities was observed when using higher-quality vector preparations for co-transductions in comparison to lower-quality preparations of AAV vectors (Figure 3B). The comparison of dual-AAV co-transduction with the full-length scar frGFP controls showed that split-inteins’ co-transductions performed with lower-quality preparations led to five-fold less fluorescence intensities. By contrast, when higher-quality vector preparations were used, the AAV vector co-transductions using split-inteins Cfa and Gp41-1 presented two-fold higher fluorescence values than that of Npu DnaE and similar fluorescence values to those of their controls (Figure 3B). The capillary Western blot analyses supported these results (Figure 3C), as lower levels of reconstituted frGFP protein were detected in co-transductions using lower-quality vector samples. Furthermore, almost two-fold less reconstituted frGFP was quantified in higher-quality AAV co-transduction using Npu DnaE, compared to the other split-inteins. The decrease in fluorescent intensities at 72 h post-transduction also correlated to a smaller amount of protein being detected (Figure S6). This can be explained by the continued cell growth after transduction. Fluorescence images also sustained these results, as they showed slight differences in fluorescence between high- and low-quality AAV vector co-transductions (Figure 3D and Figure S7).

#### 2.2.3. Decreasing Vector Doses Using Efficient Split-Inteins and Increasing Vector Quality

The above results indicate that Cfa and Gp41-1 split-inteins are more efficient in dual-AAV mediated delivery. In addition, enriched AAV vector preparations in full genome particles were demonstrated to help improve transduction. Therefore, we proposed to assess the major limitation of dual systems and to evaluate if vector doses could be reduced by combining efficient split-inteins with higher-quality vector preparations.

**Figure 4:**
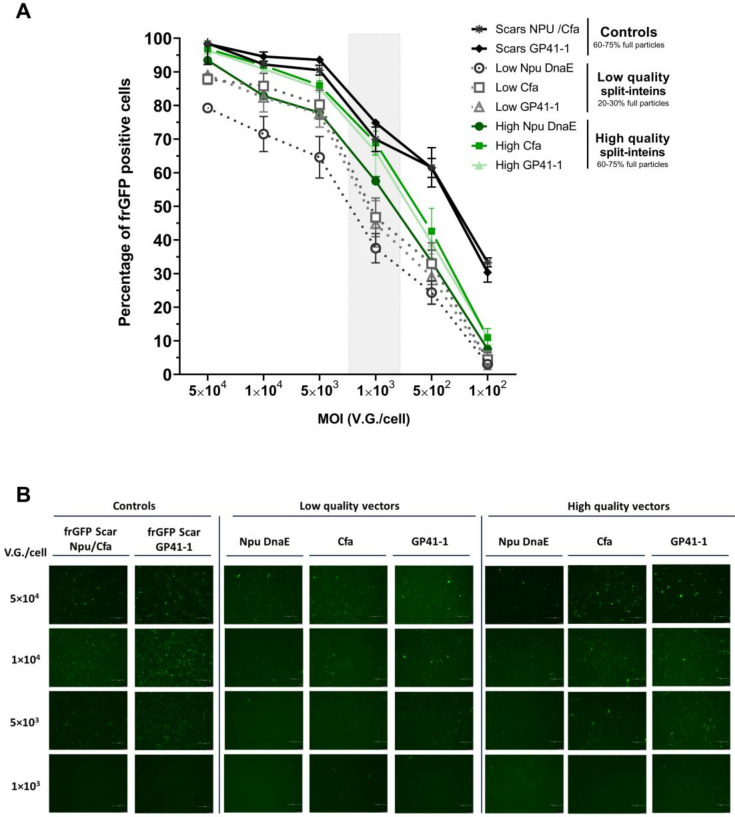
Impact of vector doses in split-inteins trans-splicing performances. Low-quality vector preparations consisted of 20–30% of full particles and high-quality preparations consisted of 60–75% of full genome particles. Both low- and high-quality dual AAV2 vector preparations were used to co-transduce HT1080 cells at six different vector doses (1 × 10^2^ to 5 × 10^4^ V.G./cell per each vector). The frGFP protein reconstitution evaluation was performed by flow cytometry at 48 h post-transduction. (**A**) Percentage of cells with reconstituted frGFP in correlation with vector dose and vector quality. The gray bar corresponds to the lowest vector dose of 1 × 10^3^ V.G./cell, where high-quality split-inteins Cfa and GP41-1 were able to achieve similar values as the control conditions with no significance and lower-quality split-inteins Cfa and GP41-1 only presented 46% of frGFP reconstitution with a significance of *p* < 0.0001 using the Tukey’s post hoc multiple comparison test. (**B**) Fluorescence images of frGFP protein expression at 48 h post-transduction. Only images from vector doses of 1 × 10^3^ to 5 × 10^4^ V.G./cell are shown, since a lack of resolution was observed in lower doses (images of all vector doses are shown in Figure S8) (scale bar = 200 μm). frGFP: folding reporter GFP. MOI: multiplicity of infection. Flow cytometry data represent the mean ± SD; *n* = 3.

For that, dual-AAV vector preparations with different vector qualities (empty and full particles) were used. Several vector doses (MOI: 5 × 10^4^ to 1 × 10^2^ V.G./cell) with up to 500-fold reduction were tested and frGFP reconstitution was measured by flow cytometry. The results showed a gradual decrease in the percentage of cells with frGFP reconstitution as the vector dose was reduced. Higher-quality preparations of Cfa- and Gp41-1-based dual-AAV vectors were able to maintain protein reconstitution to similar values as the controls, up to 1 × 10^3^ V.G./cell (around 70% positive cells). Lower-quality AAV vector preparations, however, only presented up to 46% of frGFP reconstitution (Figure 4A, Table S1 and Figure S8). The percentage of cells with frGFP reconstitution abruptly decreased from that MOI onward. Still higher frGFP percentages were always detected using the higher-quality vector preparations with Cfa and Gp41-1 split-intein dual-AAV vectors. These findings highlight that the use of dual-AAV-vectors-intein-mediated systems can be performed at lower doses when combined with the use of viral preparations enriched in full particles.

## 3. Discussion

Inteins are powerful tools for protein engineering. With the rapid developments in synthetic biology research and technology, there is a faster discovery and development of new inteins that compete with the most characterized and applied split-intein Npu DnaE [17,26,30].

Cfa and Gp41-1 are synthetically engineered and naturally occurring split-inteins, respectively, with reported higher trans-splicing rates than that of Npu DnaE [26,27]. These enhanced inteins offer an extensive potential for dual-AAV-vector-intein-mediated delivery, as they can help overcome dual-AAV vector systems’ low gene reconstitution efficiencies. Thus, the vector doses administrated can be substantially reduced. To our knowledge, only one reported study applied these split-inteins on dual-AAV vector delivery [31]. Therefore, we proposed to further evaluate their potential and to modulate favorable conditions for dual-AAV vector co-transduction.

The transient transfection of AAV vector expression cassettes allowed the first assessment of the different intein trans-splicing efficiencies without the interference of external factors, such as co-transduction inhibition. In transfection, a higher amount of each N and C-terminal was delivered to the cells, compared to viral vector transduction. Ultimately, this allowed the assessment of the split-inteins’ reconstitution efficiencies, as the availability of each terminal half, needed for protein reconstitution, was not a limiting factor. It was also important to assess whether the remaining extein amino acid residues (scars) impacted frGFP protein function. This was possible using the scar frGFP control constructs mimicking the final sequence of the protein after the trans-splicing reaction, while also serving as a control of the maximum signal of the system. Capillary Western blot results detected similar amounts of frGFP protein in all control conditions, showing that the scar residues did not affect protein production (Figure S3). In contrast, the scar frGFP controls’ fluorescence intensities decreased by two-fold when compared with those of the wild-type frGFP (Figure 1B), defining the maximum signal obtained by the split-inteins trans-splicing system. This clearly shows that the remaining extein amino acid residues left upon intein reaction had an impact on frGFP protein function. The slight change in fluorescence between the two scar frGFP controls could be justified by different frGFP interactions with the scar residues, as they vary in number and type in each construct. The impact of the split-intein residues is highly dependent on the protein of interest and on the splitting site used. Thus, a tailored screening of the protein-splitting region is needed to find the best approach without compromising the final protein function in the context of therapy. The development and use of traceless inteins is possible and highly desirable for therapeutic application. Studies that attempt to decrease the number of extein residues for efficient splicing have been designed; however, in some cases, they decrease reconstitution efficiencies [26,27]. Screening or engineering the protein-splitting region to find naturally occurring amino acids similar to the extein native residues is one strategy to achieve traceless split-inteins without compromising performance. To accurately evaluate the three split-inteins, each one was compared against the correspondent scar control. Both transient transfection and co-transduction studies (with higher-quality split-intein AAV vectors) showed a significant decrease by two-fold of protein reconstitution with Npu DnaE split-intein (Figure 1B and Figure 3A,B). Considering 48 h the peak of expression, both flow cytometry and Western blot showed that Cfa and Gp41-1 split-inteins reached the maximum of frGFP protein reconstitution possible within the system (Figure 1, Figure 3, Figures S3 and S6). Previous in vitro protein trans-splicing studies reported Cfa and Gp41-1 to be 2.5-fold and 10-fold faster than the Npu DnaE intein, respectively [26,27]. The findings reported in our study are in agreement with the findings previously described in mammalian cells, where Npu DnaE rendered the lowest efficiency, but no significant differences were observed between Cfa and Gp41-1 split-inteins [31].

Efficient dual-AAV co-transduction plays an important role in increasing the potential of protein trans-splicing. Both AAV vectors are needed to transduce the same cell to deliver the two protein halves for full-length protein reconstitution. Thus, understanding the dynamic of dual-AAV vector co-transduction and the impact of vector quality can help overcome trans-splicing limitations. In dual-AAV co-transduction, twice the amount of total viral vectors are delivered to the cells, in contrast to single transductions, thereby reaching a saturation point faster (Figure 2). This phenomenon can be due either to AAV vector competition for the cellular receptors or constraints in AAV intracellular trafficking. Some strategies can be used to overcome this, such as the use of different serotypes for each AAV vector, in order to circumvent receptor competition, or transduction enhancers, such as kinase inhibitors that have been shown to increase AAV endosomal escape [32,33,34,35,36,37]. The quality in dual-AAV vector preparation was shown to have a significant impact on split-inteins’ performances. A decrease of more than five-fold in the split-inteins’ reconstitution rates was observed when using lower-quality vector preparations (i.e., preparations with an increased number of empty or defective capsids) (Figure 3B,C). In contrast, the higher-quality vectors preparations (60–75% of full genome particles) allowed for the split-inteins to reach their full trans-splicing efficiency potential (Figure 3). The direct correlation between transduction and split-inteins’ performance is due to the shift in the availability of both N- and C-terminals needed for trans-splicing. In this work, we showed that the use of highly efficient split-inteins (Cfa and Gp41-1), in association with a three-fold increase in vector quality, enabled a decrease up to 50-fold in vector dose, while still maintaining excellent trans-splicing performances (Figure 4). The combination of more efficient split-inteins, such as the ones herein described, Cfa and Gp41-1, with higher-quality AAV vectors, allows the expansion of the potential of current dual-AAV-vector-intein-mediated systems, helping to surpass vector dose limitations and to develop more efficient therapies.

The results herein obtained establish the cornerstones for further pre-clinical studies using therapeutic genes. In vivo, co-transduction studies using a therapeutic split-gene will further expand the knowledge required to ultimately improve dual-AAV vector systems. This work shows that an efficient dual-AAV vector delivery can unblock the delivery of larger therapeutic genes, ultimately extending the use of AAV vectors in the clinic.

## 4. Materials and Methods

### 4.1. Plasmid Construction

The AAV vector expression cassette (Addgene plasmid #37825) was used as the source of AAV ITRs and as a backbone of the constructs used in this work. All double-stranded DNA sequences (gBclocks) were synthesized by Integrated DNA Technologies (Coralville, IA, USA). The backbone for all vector cassettes was developed by inserting a cDNA stuffer sequence of 1439 bp from human HPRT1 [38,39,40,41] downstream from the eGFP gene and upstream from WPRE. The pAAV2_eGFP_HPRT1 plasmid was digested at *EcoRI* and *BamHI* restriction sites to remove the eGFP gene, originating pAAV2 _HPRT1 linear backbone. A gBlock with full-length frGFP [28,29,42], followed by a GGGGS linker sequence and a myc-tag gene, were synthesized and inserted in the previously digested plasmid, ultimately creating the wild-type full-length control pAAV2_frGFP_myc_HPRT1 plasmid. The remaining constructs were carried out using the plasmid pAAV2 _HPRT1. For the split-frGFP constructs, the N-terminal gBlocks were constructed as follows: the N-terminal of the split-frGFP was fused to inteins wild-type N-extein and each N-terminal of split-intein (Npu DnaE, Cfa and Gp41-1) was followed by a GGGGS linker sequence and a 6xHis-tag. Each gBlock was inserted in the pAAV2 _HPRT1 plasmid. For the C-terminal plasmid, gBlocks were developed as follows: each C-terminal of split-intein (Npu DnaE, Cfa, and Gp41-1) was fused to its wild-type C-extein and the C-terminal of the split-frGFP was followed by a GGGGS linker sequence and a Myc-tag [26,27,28]. For the control scar constructs, full-length frGFP with the remaining intein amino acid sequences in the frGFP junction point was developed. The end of this sequence was fused to a GGGGS linker and a Myc-tag. These gBlcocks were then inserted in the pAAV2 _HPRT1 plasmid, developing the pAAV2_frGFP scar Npu/Cfa _HPRT1 and pAAV2_frGFP scar Gp41-1_HPRT1 plasmids. The pRRLSIN-mCP-GFP [43] plasmid was used to amplify by real-time qPCR the mCherry reporter gene. This sequence was inserted in the pAAV2_HPRT1, developing the pAAV2_mCherry_HPRT1 plasmid. The plasmids used for AAV vector production (pRC2 and pHelper) were commercially available from an AAV helper-free system from Agilent (Santa Clara, CA, USA).

### 4.2. Cell Lines and Culture Conditions

Human embryonic kidney 293T (HEK 293T) and HT-1080 human epithelial adherent cells, purchased from ATCC (ATCC-CRL-3216) (ATCC-CCL-121), respectively, were cultured and expanded using Dulbecco’s modified Eagle’s medium (DMEM) (# 10-013-CVR Corning™, New York, NY, USA), supplemented with 10% (*v/v*) fetal bovine serum (FBS) (Gibco™, Thermo Scientific™, Waltham, MA, USA) and maintained at 37 °C in a humidified atmosphere containing 8% CO_2_.

### 4.3. Transient Transfection Studies

Human embryonic kidney (HEK) 293T cells were seeded at 1 × 10^5^ cells/cm^2^. The cells were transfected 24 h post-seeding using PEI PRO (PolyPlus, Illkirch, France) at a mass ratio of 1:1 (DNA:PEI) and 1.5 µg of total DNA per million cells, each cassette at 0.5 µg. The medium was exchanged 24 h post-transfection to fresh DMEM with 10% (*v/v*) FBS. The reconstitution of frGFP was evaluated at different time points by flow cytometry and capillary Western blot (Jess).

### 4.4. AAV Vector Production

Production of AAV2 vectors was performed by triple transient transfection, which was an adenovirus-free production. The 293T adherent cells were seeded in ten-layer cell factories (Thermo Fisher Scientific) at 1 × 10^5^ cells/cm^2^. After seeding for 24 h, the cells were transfected with a plasmid cocktail mix in serum-free DMEM medium (Thermo Fisher Scientific), resulting in 10% of total cell factory volume. The cocktail mix contained a 1:1:1 molar ratio of pHelper:pAAV-RC2:pAAV-frGFP with 1.5 μg of total plasmid DNA per million cells and a 1:1 mass ratio of DNA to transfection reagent (PEI PRO). The medium was exchanged 24 h post-transfection to fresh DMEM with 10% (*v/v*) FBS. Cell harvest was performed at 72 h post-transfection. Following harvest, the cells were centrifuged for 10 min at 300× *g* at 4 °C and resuspended in lysis buffer composed of 20 mM Tris-HCL, pH 7.5, 150 mM NaCl, 10 mM MgCl_2_, 1% TritonX-100, 1Xprotease inhibitor cocktail (Roche, Basel, Switzerland), and 50 U/mL of Benzonase (Merck, Darmstadt, Germany). Cell lysis was carried out for 45 min at 37 °C with mild agitation. Following centrifugation for 10 min at 14,000× *g* at 4 °C, the supernatant was harvested and stored at −80 °C.

### 4.5. AAV Vector Affinity Purification and Ion-Exchange Chromatography Enrichment Step

For AAV vector affinity purification, an ÄKTA AVANT system was operated using OPUS^®^ MiniChrom^®^ Pre-packed Column with AVIPure^®^-AAV2 Affinity Resin (Repligen, Waltham, MA, USA) with 1 mL. The equilibration and washing buffers were composed of 50 mM Tris, 400 mM NaCl, 0.1% Tween 20X, pH 7.5, and the elution buffer was composed of 50 mM glycine, 150 mM NaCl, 0.1% Tween 20X, pH 2.0. Eluted pools were neutralized using 1 M Tris, 2.5 M NaCl, pH 8.0 (10% of the pool volume).

For the full capsid enrichment step, an anion exchange chromatography was developed. A similar ÄKTA system was operated using a 0.86 mL Mustang Q membrane in XT Acrodisc^®^ unit (Pall Corporation, Nova York, NY, USA). The affinity-purified sample was diluted 1:200 with 20 mM Tris, pH 8. The equilibration and washing buffers were composed of 20 mM BTP, pH 9.0, and the elution buffer was composed of 20 mM BTP, 250 mM NaCl, pH 9.0 The empty and full particles were separated by a linear separation gradient.

### 4.6. AAV2 In Vitro Transduction

HT1080 cells were seeded at 3.5 × 10^4^ cells/cm^2^, in 24-well plates. After 24 h, the cells were transduced with 200 μL of viral supernatant at a specific vector dose (MOI-multiplicity of infection) and viral supernatants were diluted using DMEM with 2% (*v*/*v*) FBS. Fresh medium was added 24 h post-transfection (DMEM with 10% (*v*/*v*) FBS). Transduction results were evaluated at different time points by flow cytometry and capillary Western blot.

### 4.7. Analytical Quantification Methods

#### 4.7.1. Total Particle Quantification

The determination of total AAV2 total particle concentration (T.P.) was performed using the AAV2 ELISA assay (Progen Biotechnik GMBH, Heidelberg, Germany) according to the manufacturer’s instructions. The absorbance was quantified at 450 nm on an Infinite PRO NanoQuant (Tecan, Männedorf, Switzerland) microplate multimode reader using a clear 96-plate well provided in the kit. The samples were applied at multiple dilutions.

#### 4.7.2. Vector Genome Copies Quantification

DNA extraction was performed according to the instructions in the High Pure Viral Nucleic Acid Kit (Roche, Basel, Switzerland) manual and was followed by real-time qPCR. Determination of the number of viral DNA copies was performed using LightCycler^®^ 480 SYBR Green I Master (Roche Applied Science, Penzberg, Germany) according to the manufacturer’s instructions on a LightCycler^®^ 480 Real Time PCR System (Roche Applied Science). The primers used were Fw (5′-ACTGTGTTTGCTGACGCAAC-3′) and Rv (5′-ACAACACCACGGAATTGTCA-3′) targeting the Woodchuck hepatitis PRE (WPRE). The reference standard used was a linear pAAV2_frGFP_myc_HPRT1 plasmid. Plasmid linearization was performed with *ScaI* enzyme within the blasticidin gene. Serial dilutions from 1 × 10^8^ copies μL^−1^ were performed.

### 4.8. Total Protein Extraction and Quantification

The cells were harvested, counted, pelleted at 300 g for 10 min. Cell lyses was performed using Mammalian Protein Extraction Reagent (M-PER) (Thermo Scientific™) with 1x cOmplete™ EDTA-free Protease Inhibitor Cocktail (Roche Applied Science), using 100 μL per 2 million of cells. The mixture was vortexed and placed at 4 °C for 10 min. Extracts were clarified by centrifugation (14.000× *g* for 10 min). Samples were stored at −20 °C for short-term and −80 °C for long-term. The total protein content was quantified using a BCA Protein Assay Kit (Thermo Fisher Scientific). Samples were applied in serial dilutions and reference material was applied in duplicate. The absorbance was measured with an Infinite PRO NanoQuant (Tecan) microplate reader.

### 4.9. Capillary Western Blot (Jess)

The Jess^TM^ Simple Western system (ProteinSimple, San Jose, CA, USA) is an automated capillary-based size separation system that is able to detect chemiluminescence and fluorescence signals. To quantify the amount of reconstituted frGFP, we followed the manufacturer’s standard method for the 12–230-kDa Jess separation module (SM-W004). In transient transfection studies, the cell extracts were diluted in PBS to a concentration of 0.5 μg/μL, and in the transduction studies the cell extracts were diluted to be at 0.8 μg/μL. The latter was provided in the kit, Streptavidin-HRP Conjugate (042–414). The primary antibodies used were myc-tag (71D10) rabbit mAb (Cell Signaling technology^®^, Danvers, MA, USA) and 6x-His tag monoclonal antibody (HIS.H8) (Invitrogen™, Waltham, MA, USA) and monoclonal anti-β-actin antibody produced in mouse (Sigma Aldrich, St. Louis, MO, USA) diluted 1:10, 1:2, and 1:10, respectively in Wes antibody diluent 2 (042–203). The secondary antibodies used were specific for this system: anti-mouse secondary antibody (042–205), anti-rabbit secondary antibody (042–206), anti-mouse NIR detection module for Jess (DM-009). The myc-tag and 6x-His tag detection was performed by chemiluminescence and β-actin by fluorescence. A digital image of the chemiluminescence and fluorescence of the capillary was captured with Compass Simple Western software 6.2.0 (Protein Simple, CA, USA) that automatically calculated heights (chemiluminescence and fluorescence intensity), area, and signal/noise ratio. The results could be visualized as electropherograms representing the peak of chemiluminescence intensity with a simulated lane view, and by using the peak area, protein quantification can also be performed.

### 4.10. Flow Cytometry

BD FACSCelesta™ (BD Bioscience, NJ, USA) was used to analyze the fluorescence of the reporter proteins (GFP and mCherry), which were present in the AAV vector expression cassette. The cells were diluted in DPBS with 2% (*v*/*v*) of FBS. Analysis of the results was performed using FlowJo software v10.9 (BD Bioscience, NJ, USA). A density plot using FSC-A and SSC-A was first applied to distinguish live and death cells. Within the live cells, a second density plot using FSC-A and FSC-H was performed to select for single cells. Single cells were than analyzed for mCherry and GFP fluorescence using the laser, yellow-green and blue, respectively. In every experiment, an internal quality evaluation of the equipment was preformed using rainbow beads to ensure that the fluorescence intensity values were comparable between experiments.

### 4.11. Statistical Analysis

The data were expressed as mean ± SD and analyzed with GraphPad Prism 8.0.1 software (San Diego, CA, USA). Statistical significance was determined using two-way ANOVA followed by Tukey’s post hoc multiple comparison test.

## Data Availability

Data is contained within the article or supplementary material.

## Supplementary Materials

The following supporting information can be downloaded at: https://www.mdpi.com/article/10.3390/ijms241310524/s1.
