# Supplementary material for: Extending AAV Packaging Cargo through Dual Co-Transduction: Efficient Protein Trans-Splicing at Low Vector Doses
Source: Int J Mol Sci. 2023 Jun 23;24(13):10524. doi: 10.3390/ijms241310524 (PMC10341399; doi:10.3390/ijms241310524)
Supplement: Supplementary file 1 [file ijms-24-10524-s001.zip › ijms-2432793-supplementary.pdf]

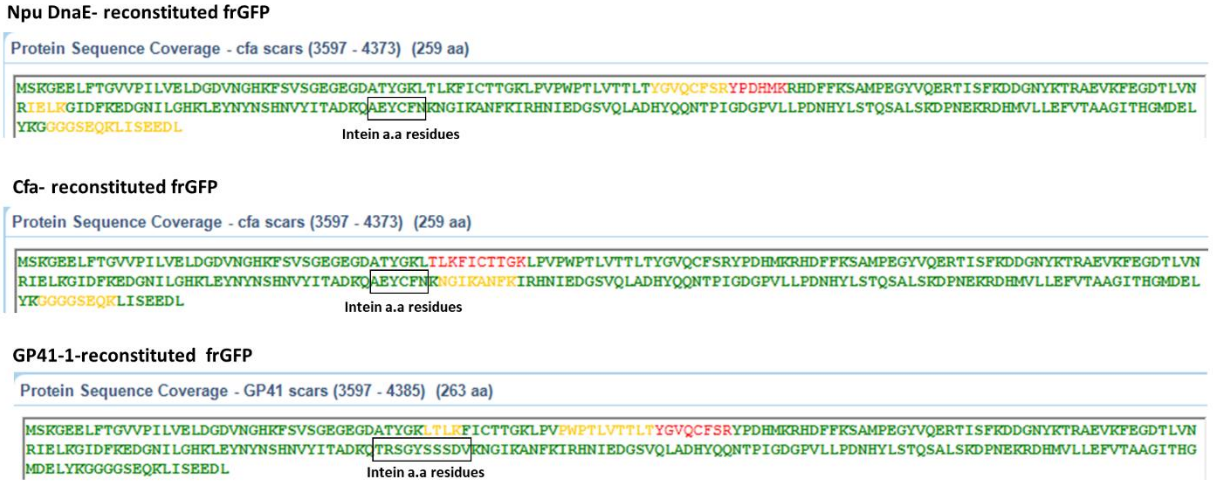

Figure S1. Peptide mapping of the final reconstituted frGFP proteins of each split-intein. Average sequence coverage of >95%. Green amino acids correspond to a peptide confidence of ≥ 95%; Yellow amino acids correspond to a peptide confidence of ≥ 50% - <90%; Red amino acids correspond to a peptide confidence of ≥ 0% - <50%. The scar amino acid residues used for the development of the control conditions were identified in the reconstituted samples, shown with the rectangle box. Peptide mapping was performed using the nanoLC-MS using Sciex TripleTOF 6600 mass spectrometer by the Mass Spectrometry Unit (UniMS), ITQB/iBET, Oeiras, Portugal.

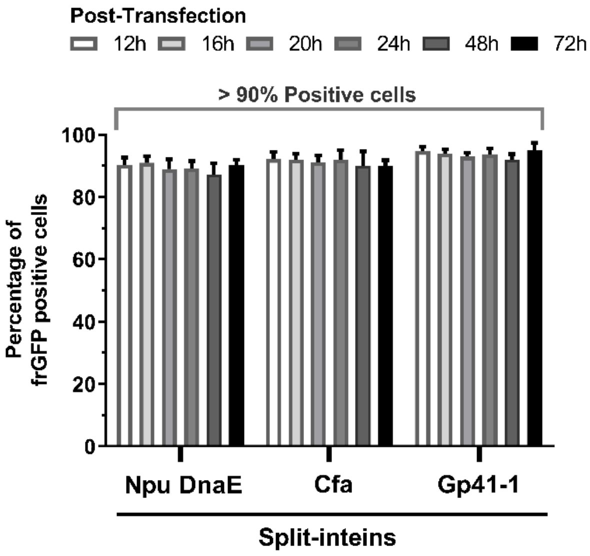

Figure S2. Split-inteins evaluation by transient transfection. Percentage of cells with reconstituted frGFP after plasmid transfection at different time points post-transfection, analysed by flow cytometry. All data represents the mean ± SD, $n = 3$.

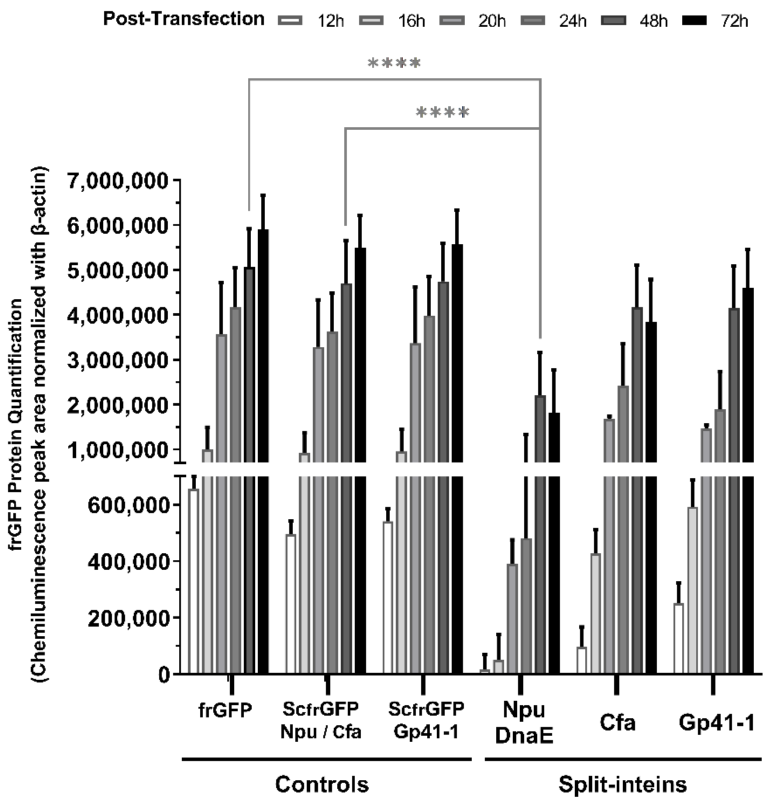

Figure S3. Split-inteins tans-splicing efficiency evaluation by transient transfection Western Blot analysis. Capillary Western blot analysis of frGFP protein reconstitution levels. Quantification of control and split-inteins frGFP protein levels at all time points post-transfection. Results are normalized to the chemiluminescence peak area of the loading control β-actin. Similar amounts of frGFP protein were detected in all control conditions. In agreement with the experimental approach as equal amounts of plasmid DNA were transfected. Cell extracts were analyzed by immunoblotting with an anti-Myc-tag antibody and anti-β-actin. Full-length or reconstituted frGFP is a 35 kDa protein detected by chemiluminescence and β-actin protein with 47 kDa by fluorescence. frGFP: folding reporter GFP. ScfrGFP: scar frGFP. All data represents the mean ± SD, n = 3; ****$p<0.0001$ by Tukey's post-hoc multiple comparison test.

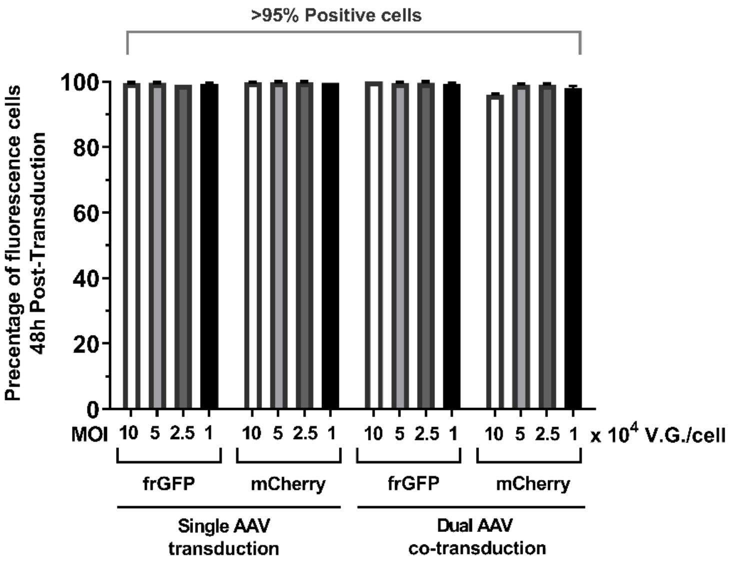

Figure S4. Evaluation of AAV2 vector single and dual co-transductions efficiencies at different vector doses. Percentage of fluorescent cells 48h post single and dual co-transduction of HT1080 cells, analysed by flow cytometry. Each bar color corresponds to the legend on the X-axis. f~~F~~rGFP: folding reporter GFP. MOI: Multiplicity of Infection. All data represents the mean ± SD, n = 3.

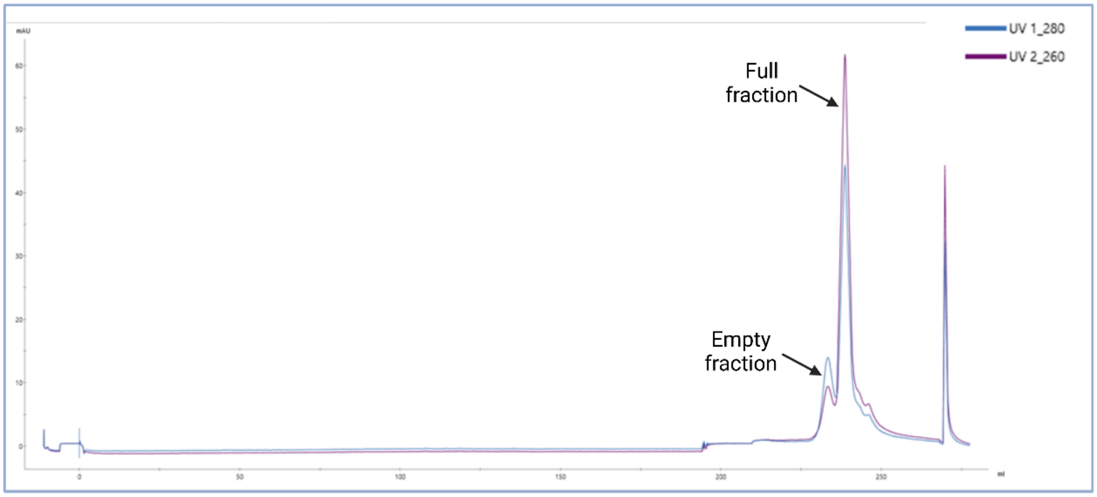

Figure S5. Representative AAV2 anion exchange chromatography elution profile. Empty and full particle separation is shown by the shift in UV absorbance profiles. Empty particle fraction presents higher absorbance at 280 nm and full particle fraction at 260 nm. The third pick observed consists of impurities, such as degraded capsids and viral DNA. For full capsid enrichment, the full fractions were harvested separately. Anion exchange chromatography was performed using a Mustang Q membrane in XT Acrodisc® unit (Pall Corporation), and a similar elution profile was obtained in all samples.

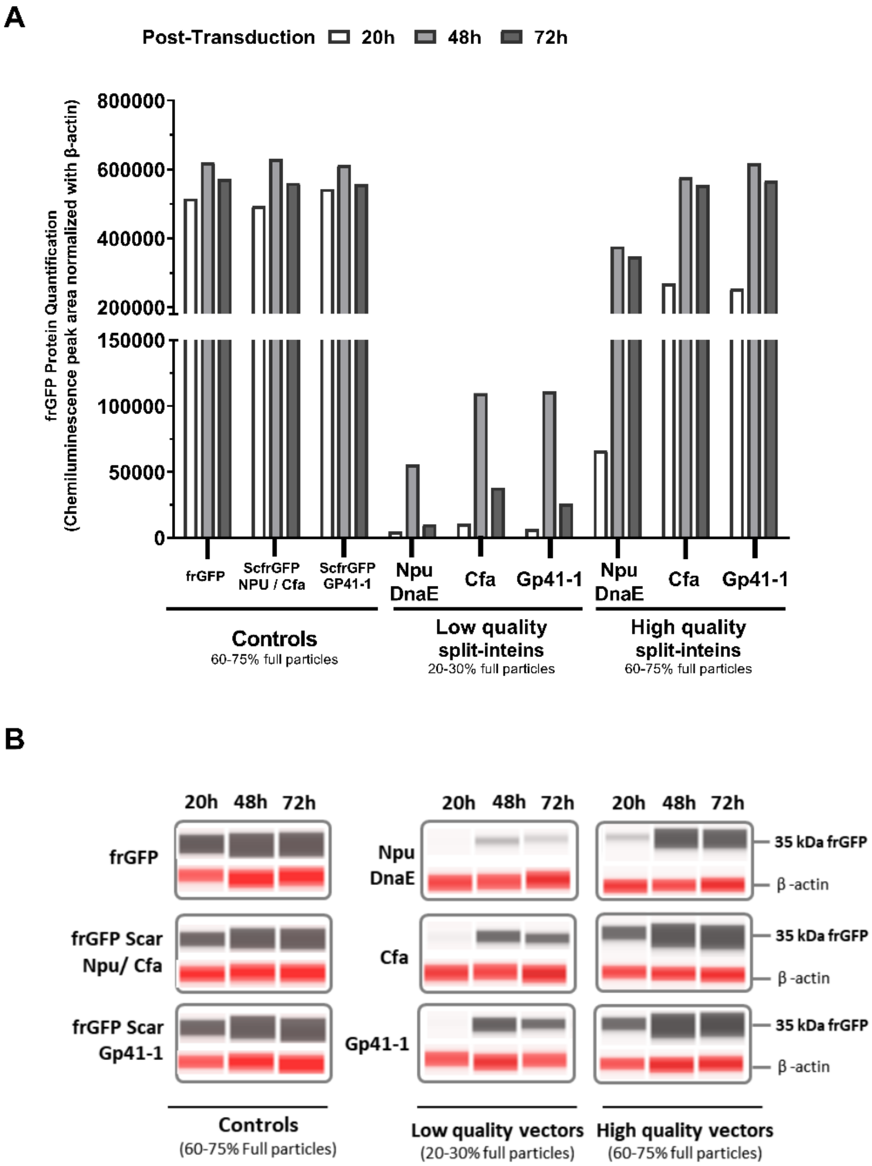

Figure S6. Impact of vector quality on split-inteins trans-splicing performances in the context of AAV dual infection analyzed by Western Blotting. Lower quality AAV vector preparations present an average of 20-30% of full particles and higher quality preparations an average of 60-75% of full particles. Capillary Western blot analysis of frGFP protein reconstitution levels after co-transduction of HT1080 cells with lower and higher quality AAV2 vector preparation using a vector dose of $5x10^4$ V.G./cell for each vector. **A.** Quantification of control and split-inteins frGFP protein levels at all time points post-transduction. Results are normalized to the chemiluminescence peak area of the loading control β-actin. Similar amounts of frGFP protein were detected in all control conditions. Cell extracts were analyzed by immunoblotting with anti-Myc-tag antibody and anti-β-actin. Full-length or reconstituted frGFP is shown as a 35 kDa protein detected by chemiluminescence and β-actin protein with 47 kDa by fluorescence. **B.** Compass for WB software simulation of a standard polyacrylamide gel run, full-length or reconstituted frGFP is observed as a 35 kDa protein. frGFP: folding reporter GFP. ScfrGFP: scar frGFP.

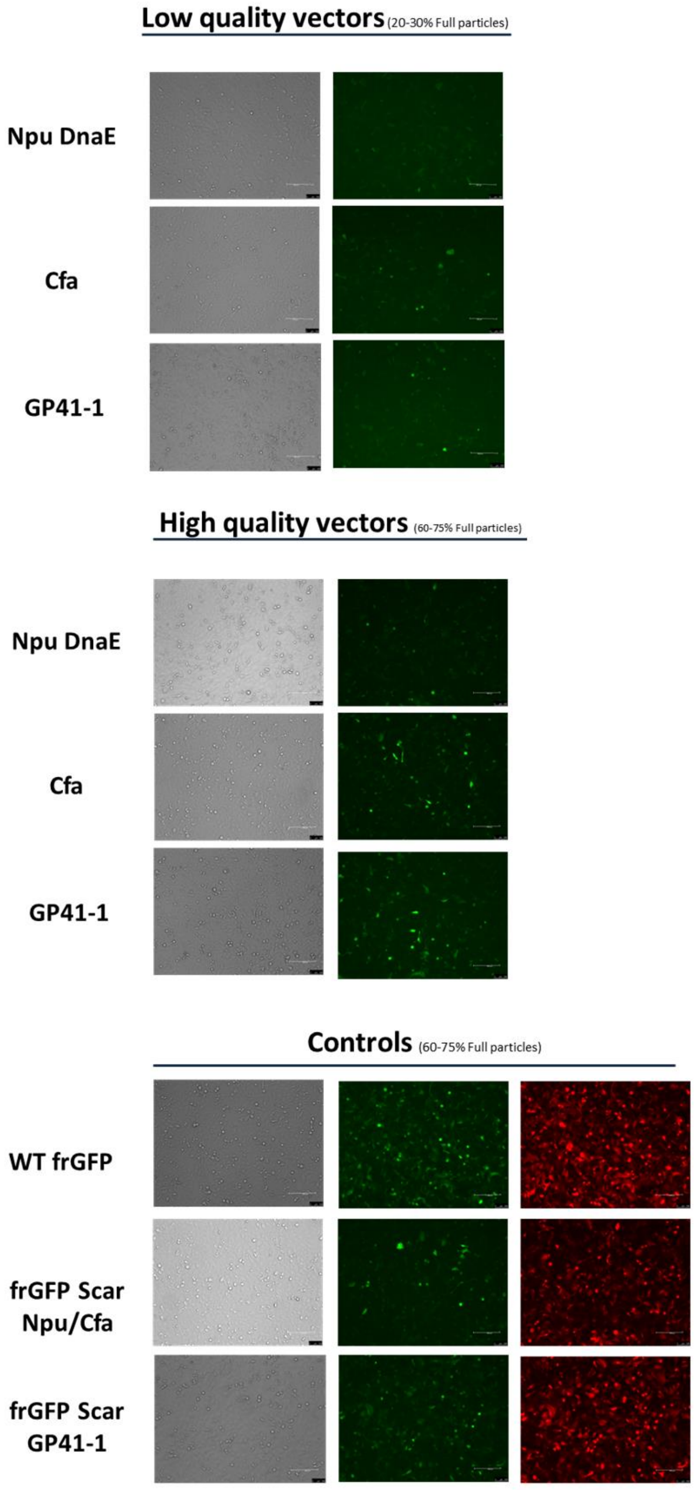

Figure S7. Impact of vector quality on split-inteins trans-splicing performances. Lower quality preparations present an average of 20-30% of full particles and higher quality preparations an average of 60-75% of full particles. Both preparations were used to co-transduce HT1080 cells with a vector dose of $5x10^4$ V.G./cell for each vector. Phase contrast and fluorescence images of frGFP protein expression and mCherry upon transduction at 48h (scale bar = 200 μm). FrGFP: folding reporter GFP.

Table S1. Impact of vector quality and split-inteins trans-splicing performances on vector doses. Percentage of cells with reconstituted frGFP.

		MOI (V.G./Cell)											
		$5x10^4$		$1x10^4$		$5x10^3$		$1x10^3$		$5x10^2$		$1x10^2$	
		% of frGFP	SD	% of frGFP	SD	% of frGFP	SD	% of frGFP	SD	% of frGFP	SD	% of frGFP	SD
Controls (60-75% full particles)	frGFP scars NPU /Cfa	98.5	0.5	92.2	0.3	90.5	1.5	70.0	3.7	61.6	5.8	33.4	1.3
	frGFP scars GP41-1	98.3	6.1	94.6	1.4	93.6	0.6	74.9	0.7	61.4	2.8	30.4	2.9
Low quality vectors (20-30% full particles)	Npu DnaE	79.3	0.8	71.5	5.2	64.6	6.2	37.6	4.4	24.4	3.5	3.0	0.8
	Cfa	87.8	1.5	85.8	3.8	80.3	4.3	46.7	5.8	33.0	6.2	4.4	2.0
	GP41-1	89.1	0.4	82.4	4.3	77.6	4.0	44.8	6.9	29.1	8.1	4.2	2.8
High quality vectors (60-75% full particles)	Npu DnaE	93.5	1.1	82.9	1.2	77.9	1.8	57.5	1.3	33.5	3.4	7.5	0.5
	Cfa	96.9	0.6	91.9	0.7	86.0	1.4	68.9	3.5	42.6	6.8	11.0	2.8
	GP41-1	96.4	2.5	90.9	3.9	85.1	4.0	66.2	8.3	39.1	0.5	11.2	2.3

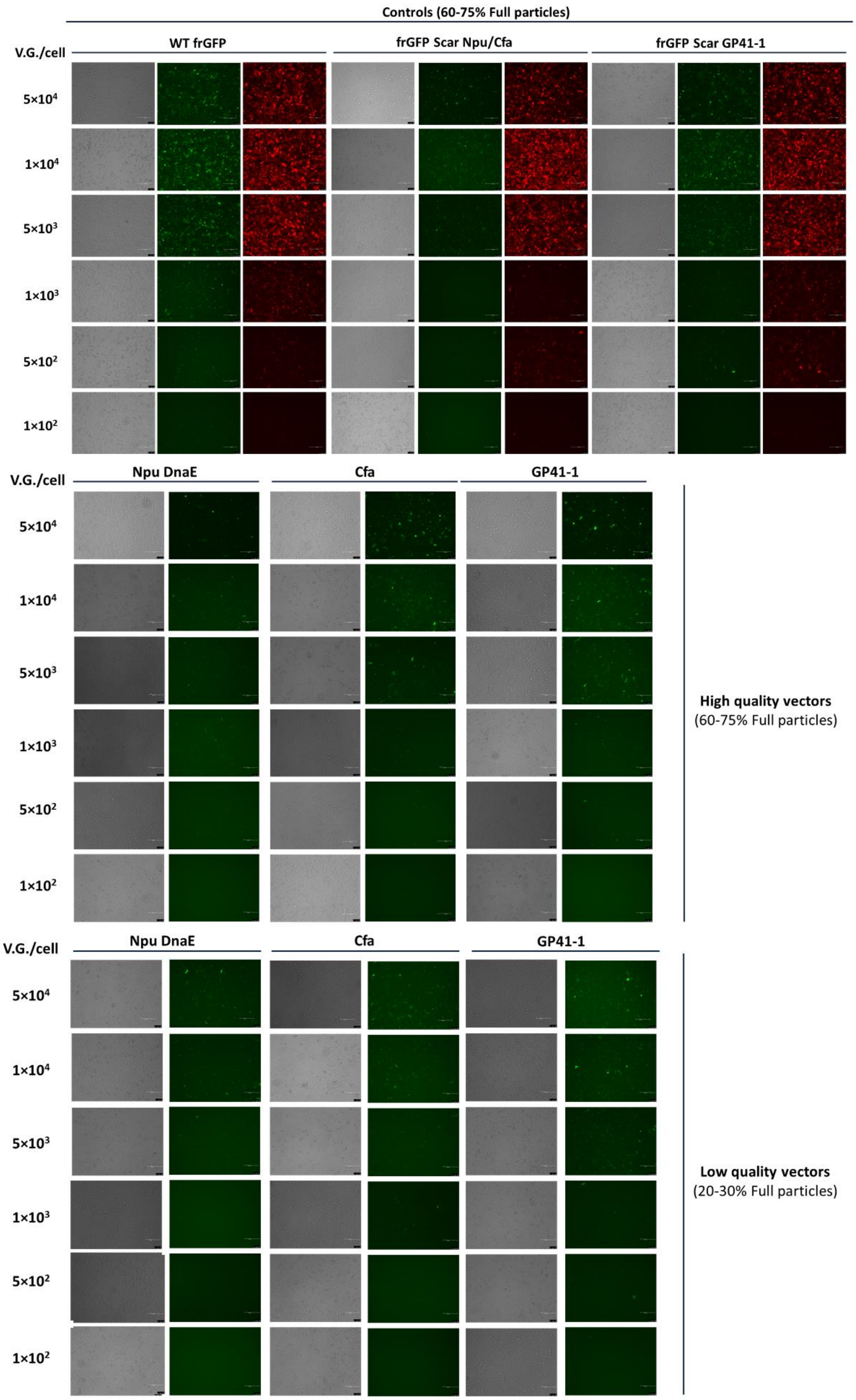

Controls (60-75% Full particles)
V.G./cell
WT frGFP
frGFP Scar Npu/Cfa
frGFP Scar GP41-1
5×10⁴
1×10⁴
5×10³
1×10³
5×10²
1×10²
V.G./cell
Npu DnaE
Cfa
GP41-1
5×10⁴
1×10⁴
5×10³
1×10³
5×10²
1×10²
High quality vectors
(60-75% Full particles)
V.G./cell
Npu DnaE
Cfa
GP41-1
5×10⁴
1×10⁴
5×10³
1×10³
5×10²
1×10²
Low quality vectors
(20-30% Full particles)

Figure S8. Impact of vector quality and split-inteins trans-splicing performances on vector doses. Low quality preparations are constituted of 20-30% of full particles and high quality preparations of 60-75% of full particles. Both preparations were used to co-transduce HT1080 cells at six different vector doses ($1x10^2$ to $5x10^4$ V.G./cell per each vector). Phase contrast and fluorescence images of frGFP and mCherry protein expression at 48h post-transduction. (scale bar = 200 μm). FrGFP: folding reporter GFP.
